# Preconception care knowledge, attitudes, and practices among women attending a private women's hospital in Riyadh, Saudi Arabia: a cross-sectional study

**DOI:** 10.3389/fgwh.2026.1947977

**Published:** 2026-09-23

**Authors:** Rola E. Alhabbash, Aiman Y. Alwadi, Sadeq B. Abu-Dawas, Abdullah Binsaeedu, Beenish Maqsud, Sally Yahya, Fouad F. Jabri, Mohammad H. Rajab, Noara K. Alhusseini

**Affiliations:** 1 Department of Biostatistics, Epidemiology and Public Health, College of Medicine, Alfaisal University, Riyadh, Saudi Arabia; 2 Department of Pharmaceutical Sciences, College of Pharmacy, Alfaisal University, Riyadh, Saudi Arabia; 3 Obstetrics and Gynecology, Women's Health Hospital, Dr. Sulaiman Alhabib Medical Group, Riyadh, Saudi Arabia; 4 Department of Global Public Health, Karolinska Institutet, Stockholm, Sweden

**Keywords:** cross-sectional study, health services accessibility, knowledge, attitudes and practices, preconception care, reproductive health, Saudi Arabia, women's health

## Abstract

**Introduction:**

Preconception care (PCC) is an important preventive strategy for improving maternal and fetal outcomes by addressing modifiable health risks before conception, yet its uptake remains limited in Saudi Arabia and evidence from private healthcare settings is scarce. This study assessed PCC knowledge, attitudes, and practices among women attending a private women's hospital in Riyadh, identified perceived barriers to PCC utilization, and examined factors associated with these outcomes.

**Methods:**

A cross-sectional questionnaire study incorporating one open-ended question on barriers was conducted among 203 women aged 18–45 years attending outpatient clinics at a private women's hospital in Riyadh during March 2025. Participants were recruited by convenience sampling and completed a structured Arabic questionnaire adapted from a previously published instrument, expert-reviewed and pilot-tested, with acceptable internal consistency for the attitude and practice scales (Cronbach's alpha 0.727 and 0.862). Scores were dichotomized using a modified Bloom's cut-off, with sensitivity analyses at alternative thresholds. Associations were assessed using chi-square or Fisher's exact tests and Firth penalized logistic regression.

**Results:**

Knowledge was high, with participants answering 92.3% of items correctly, and attitudes were predominantly favorable, with 89.2% scoring at or above the neutral midpoint. Practice was low, and 56.2% reported no preconception preparation. Knowledge and practice were not associated (*r* = −0.001), and no sociodemographic characteristic was associated with either preconception preparation or attitude. The most frequently reported barriers were lack of awareness of available services (46%), financial constraints (25%), difficulty of access (25%), and unplanned pregnancy (14%).

**Discussion:**

Favorable knowledge and attitudes were not accompanied by preventive action, and the barriers women described were predominantly structural rather than cognitive. These findings identify potentially important targets in this setting, particularly the visibility, affordability, and routine offering of preconception services. As the study was conducted in a single private facility using convenience sampling, multicenter and population-based studies are needed to establish wider applicability.

## 1 Introduction

Preconception care (PCC) is defined as the provision of biomedical, behavioral, and social health interventions to women and couples before conception occurs (1). It also refers to a set of preventive measures focused on women of reproductive age and their partners to improve pregnancy outcomes (2). The American College of Obstetricians and Gynecologists describes PCC as including screening and evaluation for genetic disorders, vaccinations, infectious diseases, behavioral and psychosocial factors, medical history, and chronic conditions (3).

Preconception care has public health value because many pregnancy-related risks arise before the first antenatal visit and may already have affected fetal development by the time pregnancy is recognised (4). Where the preconception risk profile of women of reproductive age has been measured at population level, the scope for intervention is substantial. In United States national surveillance of non-pregnant women aged 18–44 years, 21.9% reported having been told by a healthcare provider that they had a depressive disorder, 10.9% reported hypertension, 16.9% were current cigarette smokers, and only 44.9% were of normal weight (5). Among women in the same surveillance system who had recently given birth, only 33.6% reported taking a multivitamin, prenatal vitamin, or folic acid supplement every day during the month before pregnancy (5). Approximately half of pregnancies in that population are unintended, which means that risk reduction cannot reliably be deferred to a planning interval that many women never experience (6). Preconception care has been associated with improved maternal and child health through optimisation of chronic disease management, smoking cessation, nutritional support, vaccination, and other preventive interventions (7).

Equivalent population-level preconception surveillance is not available for Saudi Arabia, but the burden of the conditions that preconception care is designed to address is well documented. In the Riyadh Mother and Baby cohort of 9,723 women, 24.2% had gestational diabetes and 4.3% had pregestational diabetes, proportions that are among the highest reported internationally. Gestational diabetes was associated with increased odds of delivering a macrosomic infant, and pregestational diabetes with increased odds of caesarean delivery, preterm delivery before 37 weeks, stillbirth, and neonatal intensive care admission (8).

One of the most widely recognised preconception interventions is folic acid supplementation, which reduces the risk of neural tube defects (9). Awareness of the correct timing of supplementation, however, remains limited among Saudi women. In a cross-sectional study of 613 women of childbearing age in Saudi Arabia, only 25.3% identified the period before pregnancy as the correct time to begin folic acid, whereas 37.0% believed the first trimester was appropriate (10). Among 600 Saudi women aged 18–45 years attending outpatient clinics in Riyadh, awareness that folic acid prevents birth defects was high, yet a substantially smaller proportion reported taking it at the recommended time (11). Preventive opportunities are therefore being missed during the interval in which they would be most effective.

Saudi Arabia has established infrastructure relevant to preconception health, although no national programme or clinical guideline currently addresses preconception care as such. The most developed component is the national Premarital Screening and Genetic Counselling Programme, delivered by the Ministry of Health and now known as the Healthy Marriage Program. Introduced in 2004 to screen prospective couples for sickle cell disease and *β*-thalassemia, it was extended in January 2008 to include mandatory testing for human immunodeficiency virus and for hepatitis B and C viruses as a prerequisite for issuing a marriage certificate (12, 13). The programme operates at national scale. In the Riyadh region alone, 916,295 individuals were screened during 2021 and 2022 (14). It has also demonstrably influenced reproductive decisions. Over its first six years, the prevalence of *β*-thalassemia among examined persons fell from 32.9 to 9.0 per 1,000, the frequency of at-risk couples fell by approximately 60% from 10.1 to 4.0 per 1,000, and voluntary cancellation of marriage proposals among at-risk couples rose from 9.2% to 51.9% (12). Ministry of Health guidance advises couples to attend at least three months before the intended marriage date, and the resulting compatibility certificate is valid for six months (15).

Beyond this programme, services relevant to preconception health are delivered opportunistically rather than through a defined pathway. Ministry of Health primary healthcare centers provide chronic disease management, immunization, and reproductive health counselling. Obstetric and gynecological clinics in both the governmental and private sectors provide contraceptive, fertility, and pre-pregnancy advice on request. At policy level, the Health Sector Transformation Programme under Vision 2030 has made disease prevention an explicit strategic objective and has introduced a new Model of Care organized around six service domains, two of which, Keep Well and Safe Birth, encompass the territory that preconception care occupies (16, 17). What is not specified at national level is the preconception pathway itself, meaning which women should be offered preconception care, at which routine healthcare contacts, by which cadre of professional, and with what content. Structured preconception risk assessment is therefore concentrated at a single point in the reproductive life course, the premarital visit, which reaches couples once, at the time of marriage, and is directed at genetic and transmissible disease risk rather than at the chronic, nutritional, and behavioral risks that dominate the preconception literature. Outside that visit, risk assessment tends to occur during antenatal care, by which time several interventions can no longer achieve their full effect.

The Saudi evidence base on preconception care is small and concentrated in a narrow range of settings. Edris et al. surveyed 788 women aged 18–45 living in Saudi Arabia, recruited online through social media and excluding women working or studying in the medical field, and reported high knowledge in 44.2% (4). Sabr et al. surveyed women attending a governmental university tertiary hospital in Riyadh (18). Mousa et al. assessed awareness and utilization among women in Al Ahsa (19). Two further studies address the supply side rather than women themselves. Alamer et al. conducted a phenomenological study based on in-depth interviews with primary healthcare physicians in Ministry of Health centers in Riyadh (2), and Fussi and Mandoura surveyed general practitioners, family physicians, practitioner nurses, and midwives in primary healthcare centers in Jeddah, reporting that no written protocol for preconception care exists and that it is not routinely practiced in Saudi Arabia (20). Alomair et al. explored sexual and reproductive health perceptions qualitatively, without a specific preconception focus (21).

Several limitations recur across this literature and constrain what can be concluded from it. First, no study has been conducted in a private healthcare facility. The facility-based studies were carried out in governmental or academic tertiary institutions, and the largest study of women was conducted entirely online. Second, recruitment has been by convenience or through social media, which precludes population-level prevalence estimates and tends to over-represent women who are digitally connected and more highly educated. Third, the instruments differ between studies and the thresholds used to define good knowledge or good practice are typically derived from each study's own sample distribution rather than from clinical or theoretical criteria, so reported prevalences are not directly comparable across studies. Fourth, two of the Saudi studies describe provider perspectives, which document supply-side constraints but say nothing about what women themselves know or do. Fifth, practices and perceived barriers are reported less consistently than knowledge and attitudes, and barriers are rarely elicited from women in their own words. Taken together, what remains unknown is how women who obtain care outside the governmental system understand and act on preconception health, and what they themselves identify as preventing them from obtaining it.

Two features of the Saudi healthcare system make the private sector a useful setting in which to examine preconception care. First, private provision is substantial rather than marginal. Compulsory employment-based health insurance requires private-sector employers to insure both Saudi and non-Saudi employees, which routes a large share of the working population and their dependents towards private providers (22). Second, the women who use these services differ in ways that are relevant to preventive care. Research on healthcare-seeking behaviour among adults in Riyadh indicates that people with higher education and greater financial resources are more likely to choose private healthcare services (23). A systematic review across Gulf Cooperation Council countries similarly found that private health insurance uptake was positively associated with higher education and with employment at higher income, and that insurance was in turn positively associated with healthcare utilization, particularly routine check-ups and preventive services (24).

This evidence concerns healthcare-seeking and preventive service use in general rather than preconception care specifically, and it is not uniform. A study of primary care utilization in Riyadh found that patients with health insurance used primary care services less frequently than those without (25). The expectation that women attending a private women's hospital would demonstrate comparatively favourable preconception knowledge and practice is therefore treated here as a hypothesis to be examined rather than as an established finding, and the sociodemographic profile of the present sample is reported in full in the Results so that readers may judge the extent to which it holds. The setting is informative in either direction. If knowledge and practice prove to be high, the principal constraint elsewhere is likely to be access. If they remain low despite comparatively favourable education, income, and insurance coverage, then availability and affordability are not by themselves sufficient, and the deficit lies instead in how preconception care is offered, communicated, and integrated into routine healthcare contacts.

This study therefore aimed to assess preconception care knowledge, attitudes, and practices, to identify perceived barriers to PCC utilization, and to examine factors associated with these outcomes, among women aged 18–45 years attending outpatient clinics at a private women's hospital in Riyadh, Saudi Arabia. To our knowledge it is the first such assessment in a Saudi private healthcare facility, and it reports practices alongside knowledge and attitudes while eliciting barriers from women in their own words. Because this population encounters comparatively few availability and affordability constraints, the findings bear on whether the shortfall in preconception care is better understood as a problem of service availability or one of awareness and service design, a distinction that determines where interventions should be directed.

## 2 Materials and methods

### 2.1 Study design and setting

This was a cross-sectional questionnaire study incorporating one open-ended question on perceived barriers to preconception care uptake. The study was conducted at Dr. Sulaiman Al Habib Women's Health Hospital, a private women's hospital in Riyadh, Saudi Arabia, during the first three weeks of March 2025.

### 2.2 Participants and recruitment

Women aged 18–45 years attending outpatient clinics at the hospital were eligible if they were able to understand and complete a self-administered questionnaire and were willing to provide informed consent. Exclusion criteria included men, hospital staff, women outside the specified age range, individuals who were not direct healthcare recipients, and those unable or unwilling to provide informed consent. Participants were recruited using convenience sampling during the study period. Data were collected by trained healthcare staff in outpatient waiting areas. Participants were informed about the study objectives, confidentiality, and their right to withdraw at any time, and the questionnaire was completed in a private setting. Recruitment continued until the target sample size was reached. The number of women approached, and the number declining participation were not recorded, and a response rate can therefore not be calculated.

### 2.3 Sample size

The sample size was calculated using the Raosoft online sample size calculator. The source population was taken as 428 women, based on outpatient attendance statistics for the obstetrics and gynecology department at the study hospital. Assuming a 5% margin of error, a 95% confidence level, and a 50% response distribution, which yields the most conservative estimate, the required sample size was 203. No formal nonresponse allowance was applied, and 203 completed questionnaires were obtained. Because participants were recruited by convenience rather than probability sampling, the 5% margin of error should be understood as indicating the precision that would be obtained under random sampling from the source population, rather than as a property of the achieved sample. The sample cannot be assumed representative, and this is noted in the limitations.

### 2.4 Study instrument

Data were collected using a structured, self-administered Arabic questionnaire adapted from an instrument previously used among Jordanian women of reproductive age (7). The source instrument had been developed in Arabic, so no translation was required, and adaptation was confined to content. Sociodemographic items were adapted to the Saudi context, including replacement of the item on living area with one on nationality and the addition of items on number of children, monthly income, and usual source of preconception care information. The knowledge, attitude, and practice items were reduced and restructured, and a fifth section containing one open-ended question on perceived barriers was added. A full item-by-item comparison with the source instrument is provided in Supplementary Table S1. Unlike the source instrument, which restricts its practice section to married participants, the present study administered the practice items to all participants.

The adapted questionnaire was reviewed by three experts, an obstetrician-gynecologist, a public health specialist, and a biostatistician, who assessed each item for relevance, clarity, and contextual appropriateness, and items were revised accordingly. This constituted a qualitative assessment of content validity. No formal content validity index was computed, and construct validity was not assessed. Psychometric evaluation was therefore limited to expert content review, pilot testing among 10 women who were excluded from the final analysis, and assessment of internal consistency on the full study sample, reported in Section 2.5.

The final questionnaire comprised five sections. The first collected sociodemographic information, including marital status recorded as married, single, divorced, or widowed, in which single denotes never married. The second assessed knowledge using five yes or no items. The third assessed attitudes using six statements rated on a 5-point Likert scale. The fourth assessed practices using a filter question on whether any preconception preparation had been undertaken, followed by a checklist of nine behaviors. The fifth contained the open-ended barriers question. The full questionnaire, in Arabic with an English translation, is provided as Supplementary Appendix A, B.

### 2.5 Scoring and outcome classification

Knowledge was assessed using five yes or no items covering the definition, timing, and purpose of preconception care. Each correct response was scored 1 and each incorrect response 0, giving a possible total of 0–5.

Attitude was assessed using six statements rated on a 5-point Likert scale. All six statements were negatively worded, and the response scale was presented so that 1 corresponded to strong agreement and 5 to strong disagreement. Higher scores therefore denote disagreement with a negative statement, that is, a more favourable attitude, and no further recoding was applied. A mean attitude score ranging from 1 to 5 was calculated for each participant.

Practice was assessed using a question on whether any preconception preparation had been undertaken, followed by a checklist of nine specific behaviors completed by participants reporting preparation. Each behaviour was scored 1 if reported and 0 otherwise, giving a possible total of 0–9, and participants reporting no preparation were assigned a practice score of zero. The variable indicating preconception preparation is therefore equivalent to the practice score dichotomized at its lowest cut point, and was treated as the primary practice outcome rather than as an explanatory variable.

Internal consistency was assessed on the full sample of 203 participants rather than on the pilot sample. Cronbach's alpha was 0.727 for the attitude scale and 0.862 for the practice scale, both of which are acceptable. For the knowledge scale, alpha was 0.295, with item-rest correlations between 0.03 and 0.26. This reflects a pronounced ceiling effect, as between 85.2% and 98.0% of participants answered each knowledge item correctly and 70.4% answered all five correctly. Because a scale with this level of internal consistency and this restricted variance cannot support a meaningful composite score, knowledge is reported at item level as the proportion of participants answering each item correctly, and no composite knowledge score or knowledge classification was constructed.

For the attitude and practice domains, scores were analysed as continuous variables in the primary analysis and are reported as means with standard deviations and medians with interquartile ranges. This preserves the information in the original responses and avoids imposing a threshold that the instrument does not itself define. For comparability with the wider knowledge, attitudes, and practices literature, both domains were additionally dichotomized using a modified Bloom's cut-off point, under which participants scoring at or above 60% of the maximum attainable score in a domain were classified as positive or good (26, 27). Bloom's cut-off points are an established convention in this field, in which scores of 80%–100% are conventionally treated as good, 60%–79% as moderate, and below 60% as poor, with dichotomized modifications at 60% or 70% in wide use.

Because the reported prevalence of each classification depends on the threshold chosen, sensitivity analyses were conducted using the 80% Bloom threshold, the sample mean, and the sample median as alternative cut-offs. The prevalence of each classification under all thresholds is reported in Supplementary Table S2, and all bivariate and multivariable analyses were repeated under each threshold to establish whether the pattern of associations was stable, with results reported in Supplementary Table S3.

### 2.6 Statistical analysis

Data were analysed using SPSS version 20 and R version 4.4. Knowledge is reported at item level as the proportion answering each item correctly. Attitude and practice are reported as means with standard deviations and medians with interquartile ranges, alongside their dichotomized classifications. Associations between categorical variables were assessed using Pearson's chi-square test. Expected cell frequencies were inspected for every table, and where any was below five, Fisher's exact test or its Freeman-Halton extension was used. Sociodemographic differences in the continuous domain scores were examined using Mann–Whitney U and Kruskal–Wallis tests. Two outcomes were modelled, any preconception preparation and a positive attitude, with age group, nationality, education, marital status, number of children, employment, income, and health insurance as candidate variables. Health insurance was included because it governs access to private facilities and has been associated with greater use of preventive services in Gulf Cooperation Council countries (24). Marital status was analysed as married compared with not married and number of children as none, one to three, and four or more, to avoid unstable estimates from very small categories.

All models were fitted using Firth penalized likelihood, which yields finite estimates under sparse data or separation (28). Standard maximum likelihood estimates were computed for comparison and differed by no more than 0.21, indicating no separation once the corrections in Section 2.5 were applied. Odds ratios are the exponentiated coefficients reported in the same row, with reference categories stated in each table. All analyses were repeated under the alternative thresholds described in Section 2.5, and are reported in Supplementary Table S3. Open-ended responses were analysed thematically, as described in Section 2.7. A *p*-value below 0.05 was considered statistically significant. As the study was cross-sectional, all findings are reported as associations.

### 2.7 Analysis of open-ended responses

The final questionnaire item asked participants what barriers prevent women from accessing preconception care in Saudi Arabia. All 203 participants provided a response. Responses were typically brief, ranging from a single word to one or two sentences, and were analysed by content coding rather than by full thematic analysis. Coding proceeded inductively. Responses were read in full, recurring content was grouped into candidate categories, and these were consolidated into six themes. Where a response described more than one barrier, it was assigned to more than one theme, so theme frequencies sum to more than the number of respondents. Frequencies are reported as proportions of all 203 respondents. Within the accessibility theme, four subthemes were identified and are reported separately. Illustrative responses are presented for each theme in Table 8. The open-ended findings are reported alongside the quantitative results in Section 3.5 and are used in the Discussion to interpret the quantitative pattern, particularly the relationship between reported awareness of preconception care and reported awareness of preconception services.

## 3 Results

### 3.1 Participant characteristics

A total of 203 women were included in the analysis. Sociodemographic characteristics are presented in Table 1. The sample was predominantly Saudi (87.2%), married (91.1%), university educated or above (92.6%), and insured (85.7%), consistent with the private-sector setting of the study. Just over half were employed (54.7%), and 26.6% reported no personal income. Reported sources of preconception care information were led by the internet (65.0%) and family or friends (54.7%), while only 36.0% cited health professionals.

**Table 1:** Demographic and socioeconomic characteristics of participants (*n* = 203).

Variable	Category	Frequency	Percentage
Age group	18–24 years	13	6.4
	25–29 years	57	28.1
	30–34 years	63	31.0
	35–45 years	70	34.5
Nationality	Saudi	177	87.2
	Non-Saudi	26	12.8
Education level	Secondary/diploma or lower	15	7.4
	University	141	69.5
	Postgraduate	47	23.2
Marital status	Married	185	91.1
	Single	17	8.4
	Divorced	1	0.5
Number of children	None	76	37.4
	1–3	109	53.7
	4 or more	18	8.9
Employment status	Unemployed	92	45.3
	Employed	111	54.7
Monthly income	No personal income	54	26.6
	Less than 5,000 SAR	31	15.3
	5,000–9,999 SAR	31	15.3
	10,000–20,000 SAR	30	14.8
	More than 20,000 SAR	57	28.1
Health insurance	No	29	14.3
	Yes	174	85.7
Sources of PCC information	Internet	132	65.0
	Family/Friends	111	54.7
	Study Materials	32	15.8
	Health Professionals	73	36.0
	Reported more than one source	85	41.9

Multiple responses were permitted for sources of PCC information, so percentages for that variable sum to more than 100. The denominator is 203 for each row.

### 3.2 Knowledge, attitudes, and practices regarding preconception care

Knowledge of preconception care was uniformly high. Between 85.2% and 98.0% of participants answered each knowledge item correctly, 70.4% answered all five correctly, and 92.6% answered at least four (Table 2). Because of this ceiling effect and the low internal consistency of the scale, knowledge is reported at item level and was not classified or modelled.

**Table 2:** Preconception care knowledge, by item (*n* = 203).

Item	Correct, *n*	%
Preconception care means a woman caring for her health before pregnancy	199	98.0
The preconception period is about 3 months before pregnancy	190	93.6
Preconception care reduces fetal abnormalities during pregnancy	188	92.6
Preconception care means assessing risk factors for disease before pregnancy	187	92.1
Lifestyle should be adjusted when planning pregnancy	173	85.2

Mean knowledge score 4.62 of 5 (SD 0.67). All five items correct: 143 participants (70.4%). Four or more correct: 188 (92.6%). Cronbach's alpha 0.295.

Attitudes towards preconception care were predominantly favourable. The mean attitude score was 3.97 of 5 (SD 0.81, median 4.17, IQR 3.58–4.67), and 181 participants (89.2%) scored at or above the neutral midpoint. Agreement with negative statements was uncommon, with 74.9% disagreeing that preconception care has no positive impact on pregnancy outcome and 79.8% disagreeing that it is not an important health issue. The exception concerned who preconception care is for. Only 45.3% disagreed that preconception care is needed only by high-risk mothers, and 66.5% disagreed that it is needed only by women with a history of congenital anomalies (Table 3).

**Table 3:** Preconception care attitudes, by item (*n* = 203).

Statement	Mean	SD	Disagree, *n* (%)
Preconception care is not an important health issue	4.32	1.13	162 (79.8)
A dedicated preconception care clinic is a luxury service	4.22	1.04	148 (72.9)
Preconception care has no positive impact on pregnancy outcome	4.09	1.33	152 (74.9)
There is not enough time to plan to get preconception care	4.02	1.14	136 (67.0)
Only women with a history of congenital anomalies need preconception care	3.87	1.44	135 (66.5)
Only high-risk mothers need preconception care	3.31	1.38	92 (45.3)

Items scored 1 = strongly agree to 5 = strongly disagree. All statements are negatively worded, so higher scores denote a more favourable attitude. Overall mean 3.97 of 5 (SD 0.81, median 4.17, IQR 3.58–4.67). Cronbach's alpha 0.727.

Practice was markedly lower than either knowledge or attitude. The mean practice score was 1.59 of 9 (SD 2.31, median 0, IQR 0–3), and 114 participants (56.2%) reported no preconception preparation of any kind. Among the 89 participants (43.8%) reporting any preparation, the mean number of behaviors was 3.62 of 9 (median 3, range 1–9). Folic acid supplementation was the most frequently reported behaviour, undertaken by 72 participants (35.5% of the sample and 80.9% of those reporting any preparation), followed by improved diet quality (26.1%) and achieving an ideal body weight (23.2%). Screening and immunization behaviors were least frequent, with vaccination reported by 17 participants (8.4%) and testing for sexually transmitted diseases by 16 (7.9%) (Figure 1). Eight of the 17 unmarried participants reported having undertaken preconception preparation.

**Figure 1:**
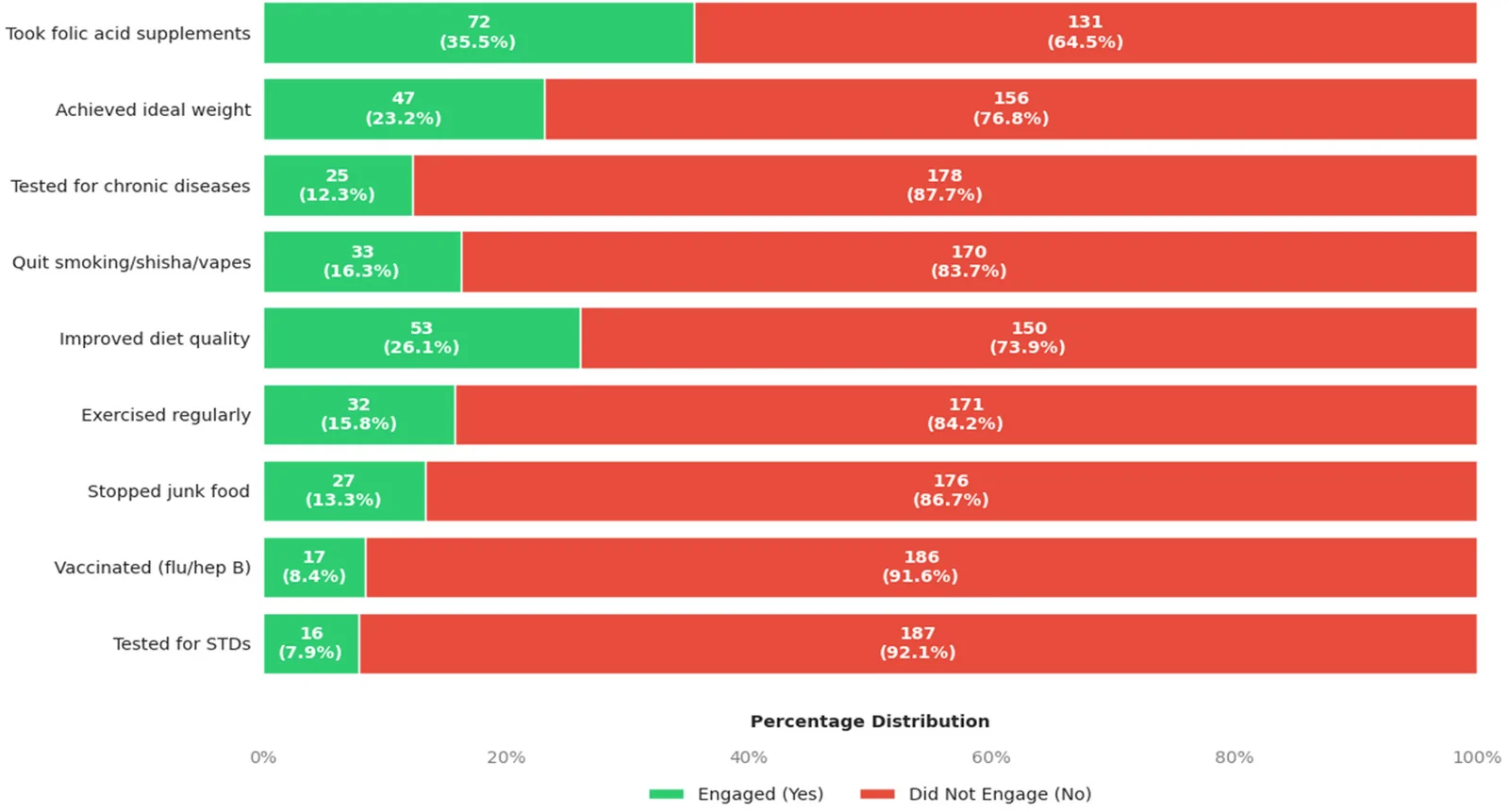
Preconception care practices reported by participants (*n* = 203).

Knowledge and practice were not associated. The correlation between the knowledge score and the practice score was r = −0.001.

### 3.3 Factors associated with attitude and preconception preparation

No sociodemographic characteristic was associated with holding a positive attitude (Table 4). Positive attitudes ranged from 83.3% among women with four or more children to 96.2% among non-Saudi participants, and no comparison approached significance. Health insurance was associated with the continuous attitude score (*p* = 0.040) but not with the attitude classification (*p* = 1.000).

**Table 4:** Positive attitude by sociodemographic characteristics (*n* = 203).

Variable	Category	Positive, n/N (%)	Test	*p*
Age group	18–24	11/13 (84.6)	Freeman-Halton	0.856
	25–29	52/57 (91.2)		
	30–34	57/63 (90.5)		
	35–45	61/70 (87.1)		
Nationality	Saudi	156/177 (88.1)	Fisher exact	0.321
	Non-Saudi	25/26 (96.2)		
Education	Secondary/diploma or lower	13/15 (86.7)	Freeman-Halton	0.873
	University	125/141 (88.7)		
	Postgraduate	43/47 (91.5)		
Marital status	Married	165/185 (89.2)	Fisher exact	1.000
	Not married	16/18 (88.9)		
Number of children	None	69/76 (90.8)	Freeman-Halton	0.660
	1–3	97/109 (89.0)		
	4 or more	15/18 (83.3)		
Employment	Unemployed	83/92 (90.2)	Chi-square	0.831
	Employed	98/111 (88.3)		
Monthly income	No personal income	48/54 (88.9)	Freeman-Halton	0.931
	Less than 5,000 SAR	27/31 (87.1)		
	5,000–9,999 SAR	29/31 (93.5)		
	10,000–20,000 SAR	26/30 (86.7)		
	More than 20,000 SAR	51/57 (89.5)		
Health insurance	No	26/29 (89.7)	Fisher exact	1.000
	Yes	155/174 (89.1)		

Positive attitude defined as a mean score of 3.00 or above, the modified Bloom 60% threshold. Exact tests were used where any expected cell frequency was below five.

Similarly, no sociodemographic characteristic was associated with having undertaken any preconception preparation (Table 5). The proportion reporting preparation ranged from 34.9% among women aged 30–34 years to 55.2% among those without health insurance, and no comparison reached significance. Analyses of the continuous attitude and practice scores using Mann–Whitney U and Kruskal–Wallis tests likewise showed no significant differences by age, nationality, education, marital status, number of children, employment, or income. Fisher's exact test or its Freeman-Halton extension was used wherever an expected cell frequency fell below five.

**Table 5:** Any preconception preparation by sociodemographic characteristics (*n* = 203).

Variable	Category	Prepared, n/N (%)	Test	*p*
Age group	18–24	6/13 (46.2)	Chi-square	0.389
	25–29	28/57 (49.1)		
	30–34	22/63 (34.9)		
	35–45	33/70 (47.1)		
Nationality	Saudi	76/177 (42.9)	Chi-square	0.641
	Non-Saudi	13/26 (50.0)		
Education	Secondary/diploma or lower	6/15 (40.0)	Chi-square	0.424
	University	66/141 (46.8)		
	Postgraduate	17/47 (36.2)		
Marital status	Married	81/185 (43.8)	Chi-square	1.000
	Not married	8/18 (44.4)		
Number of children	None	31/76 (40.8)	Chi-square	0.653
	1–3	51/109 (46.8)		
	4 or more	7/18 (38.9)		
Employment	Unemployed	41/92 (44.6)	Chi-square	0.963
	Employed	48/111 (43.2)		
Monthly income	No personal income	26/54 (48.1)	Chi-square	0.809
	Less than 5,000 SAR	15/31 (48.4)		
	5,000–9,999 SAR	14/31 (45.2)		
	10,000–20,000 SAR	11/30 (36.7)		
	More than 20,000 SAR	23/57 (40.4)		
Health insurance	No	16/29 (55.2)	Chi-square	0.260
	Yes	73/174 (42.0)		

Any preconception preparation is equivalent to a practice score of one or more.

### 3.4 Multivariable analysis

In Firth penalized logistic regression, no variable was independently associated with having undertaken any preconception preparation. All adjusted odds ratios lay between 0.58 and 1.85 (Table 6). No variable was independently associated with a positive attitude at the primary classification threshold (Table 7).

**Table 6:** Firth penalized logistic regression: any preconception preparation (*n* = 203, 89 events).

Variable	Category	β	OR	95% CI	*p*
Age group	18–24	0.170	1.19	0.30–4.66	0.808
	25–29	0.093	1.10	0.48–2.49	0.824
	30–34	−0.540	0.58	0.27–1.27	0.174
	35–45 (ref)	_	1.00	_	_
Nationality	Non-Saudi	0.300	1.35	0.53–3.42	0.527
	Saudi (ref)	_	1.00	_	_
Education	Secondary/diploma or lower	−0.321	0.73	0.23–2.30	0.586
	Postgraduate	−0.278	0.76	0.36–1.59	0.461
	University (ref)	_	1.00	_	_
Marital status	Not married	−0.116	0.89	0.29–2.72	0.839
	Married (ref)	_	1.00	_	_
Number of children	1–3	0.342	1.41	0.69–2.87	0.347
	4 or more	−0.088	0.92	0.25–3.33	0.894
	None (ref)	_	1.00	_	_
Health insurance	No	0.613	1.85	0.77–4.45	0.172
	Yes (ref)	_	1.00	_	_
Employment	Employed	0.435	1.55	0.62–3.83	0.347
	Unemployed (ref)	_	1.00	_	_
Monthly income	Less than 5,000 SAR	−0.083	0.92	0.36–2.39	0.865
	5,000–9,999 SAR	−0.194	0.82	0.26–2.59	0.740
	10,000–20,000 SAR	−0.429	0.65	0.18–2.34	0.510
	More than 20,000 SAR	−0.455	0.63	0.20–1.97	0.431
	No personal income (ref)	_	1.00	_	_

Each odds ratio is the exponentiated coefficient in the same row. Standard maximum likelihood estimates differed from the penalized estimates by no more than 0.058.

**Table 7:** Firth penalized logistic regression: positive attitude (*n* = 203, 181 events).

Variable	Category	β	OR	95% CI	*p*
Age group	18–24	−0.413	0.66	0.11–4.15	0.659
	25–29	0.287	1.33	0.40–4.47	0.642
	30–34	0.245	1.28	0.41–3.95	0.671
	35–45 (ref)	_	1.00	_	_
Nationality	Non-Saudi	1.001	2.72	0.48–15.36	0.257
	Saudi (ref)	_	1.00	_	_
Education	Secondary/diploma or lower	0.006	1.01	0.22–4.61	0.994
	Postgraduate	0.163	1.18	0.39–3.55	0.773
	University (ref)	_	1.00	_	_
Marital status	Not married	−0.261	0.77	0.16–3.77	0.748
	Married (ref)	_	1.00	_	_
Number of children	1–3	−0.369	0.69	0.24–2.03	0.501
	4 or more	−1.074	0.34	0.06–1.89	0.218
	None (ref)	_	1.00	_	_
Health insurance	No	0.166	1.18	0.34–4.16	0.796
	Yes (ref)	_	1.00	_	_
Employment	Employed	−0.738	0.48	0.13–1.77	0.269
	Unemployed (ref)	_	1.00	_	_
Monthly income	Less than 5,000 SAR	0.143	1.15	0.30–4.37	0.833
	5,000–9,999 SAR	1.261	3.53	0.57–21.84	0.175
	10,000–20,000 SAR	0.610	1.84	0.30–11.38	0.511
	More than 20,000 SAR	0.842	2.32	0.45–11.93	0.313
	No personal income (ref)	_	1.00	_	_

Standard maximum likelihood estimates differed from the penalized estimates by no more than 0.206.

In the sensitivity analyses, monthly income was associated with a positive attitude when attitude was dichotomized at the stricter 80% threshold, with adjusted odds ratios of 3.04 (95% CI 1.10–8.35) for women earning below 5,000 SAR rising to 6.41 (95% CI 1.58–26.06) for those earning 10,000–20,000 SAR, relative to women reporting no personal income. This association was not present at the primary threshold, nor in the analysis of the continuous attitude score (*p* = 0.284), and it should therefore be regarded as threshold-dependent rather than robust. Postgraduate education was similarly associated with a positive attitude at the median threshold only (OR 2.15, 95% CI 1.01–4.58). By contrast, no variable was associated with preconception preparation at any threshold examined (Supplementary Table S3).

### 3.5 Perceived barriers

All 203 participants responded to the open-ended question on perceived barriers. Percentages are calculated on that denominator, and responses could be assigned to more than one theme where more than one barrier was described. Six themes were identified (Table 8). Lack of awareness was the most frequently reported barrier, reported by 46% of participants. Participants described being unaware that preconception services existed at all, one noting that this was the first occasion on which she had learned that clinics specialize in this area, after eleven years of marriage. Financial constraints were reported by 25%, with participants describing hospital charges as prohibitive despite 85.7% of the sample holding health insurance. Accessibility was reported by 25%. Within this theme, appointment delays were most frequently described (10%), followed by geographic distance (7%), the absence of dedicated preconception services (5%), and transport difficulties (2.5%). Unplanned pregnancy was reported by 14%, described as sudden or unintended, indicating that preconception preparation had not been possible. Sociocultural factors were reported by 10%, including the perception that seeking preconception care implies an underlying problem. Informational gaps formed a minor additional theme, with participants describing an absence of reliable sources and consequent reliance on family and friends. This is consistent with the reported information sources, in which 54.7% cited family or friends while only 36.0% cited health professionals.

**Table 8:** Perceived barriers to preconception care (*n* = 203).

Theme	%	Illustrative response
Lack of awareness	46	This is the first time I know clinics specialize in this subject. I've been married 11 years.
Financial constraints	25	Prices of some hospitals are very high.
Accessibility	25	Waiting months to see a doctor.
Appointment delays	10	Exhaustingly long queues at clinics.
Geographic distance	7	Difficulty accessing specialized centers due to remote locations.
No dedicated services	5	There are no clinics focused on preconception care.
Transport difficulties	2.5	Road congestion hinders hospital access.
Unplanned pregnancy	14	I didn't even know I was at risk. It just happened.
Sociocultural factors	10	Seeking care implies a problem.

Percentages are proportions of all 203 respondents. Responses could be assigned to more than one theme, so percentages sum to more than 100. Accessibility subthemes are subsets of that theme and do not sum to it, as some responses cited accessibility without specifying a type.

## 4 Discussion

### 4.1 Principal findings

This study found high preconception care knowledge and predominantly favorable attitudes among women attending a private women's hospital in Riyadh, alongside low uptake of preconception behaviors. Participants answered 92.3% of knowledge items correctly and 70.4% answered all five correctly, while 89.2% scored at or above the neutral midpoint of the attitude scale. By contrast, 56.2% reported no preconception preparation of any kind, and among those who prepared the median number of behaviors was three of nine. Knowledge and practice were not associated (r = −0.001), and no sociodemographic characteristic was associated with either preconception preparation or attitude. This pattern is inconsistent with the assumption, implicit in much preconception health education, that raising awareness will of itself produce preventive behavior. Because all domains were measured simultaneously, no temporal or causal direction can be inferred, and we make no claim that knowledge shapes or fails to shape behavior. What these data show is that the two did not vary together in this sample. The barrier responses explain an apparent contradiction. Lack of awareness was the most frequently reported barrier, cited by 46% of participants, which appears inconsistent with the high knowledge scores until the distinction between knowledge of the concept and knowledge of the service is drawn. Participants understood what preconception care is but were unaware that it was available to them, as reflected in one response noting this was the first occasion on which the participant had learned that clinics specialize in this area, after eleven years of marriage. The remaining barriers were structural rather than cognitive, comprising cost (25%), accessibility (25%), and unplanned pregnancy (14%).

### 4.2 Comparison with previous studies

Direct comparison of these proportions with previous studies is not straightforward. Reported prevalences of good knowledge or practice depend heavily on the threshold each study applies, and those thresholds differ. Edris et al. defined high knowledge as a score above the fiftieth percentile of its own sample and reported 44.2% (4). Sabr et al. reported 51.9% with good knowledge and 45.2% with good practice at a governmental tertiary university hospital in Riyadh (18), and Alkhatib et al. reported 48.6%, 48.1% and 47.0% among Jordanian women (7). Because these figures index each sample against its own distribution or against differing criteria, a higher reported percentage does not necessarily indicate a better-informed population. We therefore compare patterns rather than prevalences, and report knowledge at item level for the same reason. Where comparison is meaningful, the differences are informative. Alkhatib et al. reported positive correlations between knowledge, attitude and practice, whereas we found none (7). Several differences may bear on this. That sample was recruited online nationally and comprised 1,368 women, whereas ours was recruited in person from a single private facility. Its practice section was restricted to married participants, so its practice measure reflects women for whom pregnancy is a realistic prospect, while ours was not. The instruments also differ in composition, since our adaptation retained five of the source knowledge items and nine of its fifteen practice items, and reduced its attitude scale to negatively worded statements only. The comparison with the governmental tertiary setting studied by Sabr et al. is of particular interest given our private-sector focus (18). Our participants had higher educational attainment and near-universal insurance coverage, yet reported low preconception practice. This does not support the expectation, set out in the Introduction, that greater healthcare access and socioeconomic advantage would translate into greater preconception uptake. Our finding that education was unrelated to knowledge is consistent with Alkhatib et al., who reported no effect of educational level on preconception knowledge, although a United Kingdom study found lower education linked to poorer knowledge (7, 29). This suggests that the gap concerns exposure to preconception-specific information rather than general educational capacity.

### 4.3 Pregnancy intention and the interpretation of low practice

Pregnancy intention was not measured in this study. Participants were women of reproductive age attending outpatient clinics for any reason, not women actively planning pregnancy. Several assessed behaviors, including folic acid supplementation, weight optimization and preconception screening, presuppose an intention to conceive, so part of the low prevalence observed is likely to reflect sample composition rather than inaction among women planning pregnancy. The barrier responses indicate this is not the whole explanation. Unplanned pregnancy was reported by 14% of participants, which is itself evidence that the opportunity for preconception preparation is frequently absent rather than declined, consistent with the observation that risk reduction cannot be deferred to a planning interval many women never experience (6). Preparation did not differ by parity (40.8% of nulliparous participants compared with 46.8% of those with one to three children, *p* = 0.653) or between married and unmarried participants (43.8% and 44.4%). Since the source instrument restricted these items to married participants and we did not, some participants may have interpreted them as general health behaviors. The practical implication is that preconception care cannot be delivered only to women who present as planning a pregnancy.

### 4.4 Evidence that preconception intervention is effective

The case for strengthening preconception provision does not rest on inference from cross-sectional surveys. At the level of outcomes, a systematic review and meta-analysis of 25 studies including 5,903 women found that preconception care reduced congenital malformations by 71% (RR 0.29, 95% CI 0.21–0.40; high-certainty evidence) and lowered first-trimester HbA1c by 1.27% (30). Earlier analyses by the same group reported reductions in perinatal mortality (RR 0.34, 95% CI 0.15–0.75) and preterm delivery (RR 0.70, 95% CI 0.55–0.90) (31). These concern maternal and child health outcomes rather than knowledge scores, and are directly relevant given the prevalence of diabetes in Saudi pregnancies (8). At the level of awareness and behavior, a review of national folic acid campaigns found preconception awareness rose from 6% to 41% following campaigning (32), and randomized preconception counselling increased the proportion of women knowing that folate prevents birth defects from 27% to 46% (RR 1.72, 95% CI 1.32–2.23) (33). A stepped-wedge cluster randomized trial in four Dutch municipalities found adherence to preconception lifestyle recommendations improved where intervention reach was greatest (RR 1.57, 95% CI 1.11–2.22) (34). Two qualifications matter here. The same campaign review found more than half of women still did not take folic acid as recommended afterwards (32), so awareness campaigns alone are insufficient, which is consistent with the present findings. And Saudi Arabia has its own demonstration that a structured national program can change reproductive decisions, since the Premarital Screening and Genetic Counselling Programme raised voluntary cancellation of at-risk marriage proposals from 9.2% to 51.9% over six years (12). The relevant precedent is therefore domestic, and involved a mandated service pathway rather than communication alone.

### 4.5 Implications for policy and practice

In a population that understood preconception care, valued it, and faced few affordability or access constraints, uptake remained low and the barriers described were predominantly structural. This locates the problem in service design rather than health education alone, and raises four questions any Saudi preconception strategy must answer.

**To whom**: Because 14% of participants described their pregnancy as unplanned and preparation did not differ by parity or marital status, a strategy directed only at women presenting as planning a pregnancy will miss many who would benefit. The appropriate target is all women of reproductive age in contact with health services, with intensified provision for those with diabetes, obesity, or a family history of genetic disease.

**At which contacts**: Saudi Arabia already possesses the necessary contact points. The Premarital Screening and Genetic Counselling Programme reaches essentially all couples entering marriage but is confined to genetic and transmissible disease risk (12, 13). Extending its counselling component to cover folic acid timing, weight, chronic disease optimization and immunization could add preconception content to a contact that already occurs. Primary healthcare centers, obstetric and gynecological clinics, and postpartum and contraceptive consultations offer further opportunities.

**By whom**: Only 36.0% of participants cited health professionals as an information source, while 54.7% cited family or friends. Saudi primary care physicians have identified time constraints and role ambiguity as obstacles (2), and primary care staff in Jeddah report that no written protocol exists and preconception care is not routinely practiced (20). Provision therefore depends on whether a cadre is designated, trained and resourced.

Provision in the private sector requires different mechanisms, since private providers operate outside Ministry of Health programme structures and preconception content cannot be mandated through them. The most direct lever is financing. Cooperative health insurance schemes could specify preconception consultation as a covered benefit, which would address the cost barrier reported by 25% of participants despite 85.7% holding insurance. Given the continuity of care typical in these settings, a brief structured prompt at existing appointments may also be more feasible than a dedicated preconception clinic.

**With what content:** Only 45.3% of participants disagreed that preconception care is needed solely by high-risk mothers, and 66.5% that it is needed solely by women with a history of congenital anomalies. Preconception care is widely understood here as remedial care for identified risk rather than universal prevention, and messaging addressing this directly is likely to be more useful than restating that preconception care is beneficial.

Two structural elements complete the picture. Beyond insurance coverage, the absence of a national preconception guideline means no document currently specifies any of the four elements above, and the Health Sector Transformation Program's model of care provides an existing framework within which such a pathway could be defined (16, 17).

### 4.6 Strengths and limitations

The principal strength of this study is its setting. Women receiving care in private facilities are absent from the Saudi preconception literature, and studying a population facing few availability or affordability constraints allows the observed shortfall to be attributed with more confidence to service design. The study also reports practices alongside knowledge and attitudes, and elicits barriers in women's own words. The persistence of low practice here indicates that underuse is not confined to traditionally underserved populations. Several limitations should be considered when interpreting these findings.

**Design and sampling**: The cross-sectional design precludes causal or temporal inference. Participants were recruited by convenience sampling from a single private hospital over three weeks, so the sample is not population-based and cannot be generalized to women in Riyadh or Saudi Arabia. The number approached and the number declining were not recorded, so no response rate can be calculated. Self-administered questionnaires are subject to recall and social desirability bias.

**Measurement**: No formal content validity index was computed and construct validity was not assessed. The five-item knowledge scale showed low internal consistency (alpha 0.295) and a pronounced ceiling effect, partly attributable to conversion of the source instrument's multiple-choice items to a yes or no format, and knowledge is therefore reported at item level. The adapted attitude scale retained only negatively worded statements, removing the polarity check a balanced scale provides; future adaptations should preserve a mixture. The junk food item was not defined, so responses reflect participants' own interpretation. Health insurance was recorded as a binary variable whereas the source instrument distinguished private from governmental coverage.

**Classification**: Attitude and practice were dichotomized using a modified Bloom's cut-off, an established convention that is not clinically derived, and misclassification near the threshold is possible. Sensitivity analyses appear in Supplementary Tables S2, S3, and the association between income and positive attitude was present only at the stricter thresholds and not in the continuous analysis, so should be regarded as threshold-dependent. No variable was associated with preconception preparation at any threshold examined.

**Analysis**: Several contingency tables contained sparse cells, and exact methods were used where chi-square assumptions were not met. Models were fitted using Firth penalized likelihood and small categories collapsed. Some subgroups nonetheless remained small, and the study was not powered to detect modest associations, so the absence of significant associations should not be read as evidence that none exists.

**Scope**: Pregnancy intention was not measured, and the practice items were administered to all participants rather than to married participants only, as in the source instrument. The qualitative component consisted of a single open-ended question, which limits the depth of the barrier findings, and theme frequencies are reported as proportions of all respondents.

These proposals are offered as directions warranting evaluation rather than established requirements. The findings identify where preconception provision appears to fail in one well-resourced setting, and cannot establish the national distribution of these barriers or their relative weight. Multicenter studies spanning private and governmental facilities, and population-based sampling, would be required to determine how far this pattern extends. Studies incorporating a measure of pregnancy intention, and evaluations of whether extending preconception content to existing service contacts changes uptake, would be of particular value.

## 5 Conclusions

Among women attending a private women's hospital in Riyadh, knowledge of preconception care was high and attitudes were predominantly favorable, yet more than half reported no preconception preparation of any kind. Knowledge and practice were not associated, and no sociodemographic characteristic was associated with either preconception preparation or attitude. The barriers participants described were predominantly structural, comprising unawareness that services exist, cost, difficulty of access, and unplanned pregnancy, rather than a deficit in understanding or motivation. These findings identify potentially important targets for improving preconception care among women attending this private healthcare setting, particularly the visibility, affordability, and routine offering of preconception services at healthcare contacts women already attend. Because the study was conducted in a single private facility using convenience sampling, multicenter and population-based studies are needed to determine the extent to which these findings apply more broadly in Saudi Arabia.

## Data Availability

De–identified data supporting the conclusions of this article may be made available on reasonable request, subject to approval by the Institutional Review Board of Dr. Sulaiman Al–Habib Medical Group Research Center. Requests should be directed to Aiman Y. Alwadi, aalwadi@alfaisal.edu.
