# Supplementary material for: Preconception care knowledge, attitudes, and practices among women attending a private women's hospital in Riyadh, Saudi Arabia: a cross-sectional study
Source: Front Glob Womens Health. 2026 Sep 23;7:1947977. doi: 10.3389/fgwh.2026.1947977 (PMC13642619; doi:10.3389/fgwh.2026.1947977)
Supplement: Supplementary file 1 [file Table1.docx]

**Supplementary Table S1.** Item-by-item comparison with the source instrument.

| **Present study** | **Source** | **Modification** |
| --- | --- | --- |
| **1. Knowledge** | | |
| PCC means a woman caring for her health before pregnancy | Demographic Q8, option a | Split from multiple choice into standalone yes/no item |
| PCC means assessing risk factors for disease | Demographic Q8, option b | Split from the same item |
| The preconception period is about 3 months | Knowledge Q2, option c | Converted from multiple choice to yes/no |
| PCC reduces fetal abnormalities | Knowledge Q1, option c | Converted from multiple choice to yes/no |
| Lifestyle should be adjusted when planning | Practice item 4 | Relocated from the practice section |
| **2. Attitude** | | |
| PCC has no positive impact on pregnancy outcome | Attitude 1, first clause | Compound statement separated |
| PCC is not an important health issue | Attitude 1, second clause | Compound statement separated |
| A dedicated PCC clinic is a luxury service | Attitude 2 | Retained with rewording |
| There is not enough time to plan to get PCC | - | Newly written for this study |
| Only high-risk mothers need PCC | Attitude 5, first clause | Compound statement separated |
| Only women with a history of anomalies need PCC | Attitude 5, second clause | Compound statement separated |
| - | Attitude 3, 4 and 6 | Not retained |
| **3. Practice** | | |
| Folic acid 3 months before conception | Practice 1 | Retained |
| Achieved ideal body weight | Practice 3 | Retained |
| Tested for chronic diseases | Practice 6 | Compound item separated |
| Quit smoking, shisha or vaping | Practice 8 | Shisha and vaping added |
| Improved diet quality and diversity | Practice 9 | Retained |
| Exercised regularly, 2 to 5 times weekly | Practice 10 | Frequency specified |
| Stopped consuming junk food | Practice 12 | "Ready-to-eat foods" reworded |
| Vaccinated, influenza or hepatitis B | Practice 4 | Retained |
| Tested for sexually transmitted diseases | Practice 6 | Compound item separated |
| - | Practice 2, 5, 7, 11, 13, 14, 15 | Not retained |
| **4. Sociodemographic** | | |
| Living place | Three governorate regions | Replaced with nationality |
| Education | Five categories | Collapsed to three |
| Marital status | Married, single, other | Expanded to four categories |
| Employment | No, full-time, part-time | Collapsed to binary |
| Health insurance | No, private, governmental | Collapsed to binary |
| Monthly income | Not present | Added, five categories |
| Number of children | Three categories | Expanded to four |

**Supplementary Table S2.** Prevalence of positive attitude and good practice under alternative classification thresholds (n = 203)

| **Domain** | **Bloom 60%** | **Bloom 80%** | **Sample mean** | **Sample median** |
| --- | --- | --- | --- | --- |
| Positive attitude | ≥3.00, 181 (89.2%) | ≥4.00, 116 (57.1%) | ≥3.97, 116 (57.1%) | ≥4.17, 103 (50.7%) |
| Good practice | ≥5.4, 19 (9.4%) | ≥7.2, 5 (2.5%) | ≥1.59, 72 (35.5%) | Not applicable (median = 0) |
| Knowledge (not classified) | ≥3.0, 201 (99.0%) | ≥4.0, 188 (92.6%) | ≥4.62, 143 (70.4%) | ≥5.0, 143 (70.4%) |

Knowledge is shown for information only. It was not classified, owing to a Cronbach's alpha of 0.295 and a pronounced ceiling effect. In the attitude model, monthly income was significantly associated with a positive attitude only at the stricter thresholds (adjusted OR 3.04-6.41). No association was present at the primary threshold or in analysis of the continuous score (p = 0.284).

**Supplementary Table S3.** Factors significantly associated with each outcome under alternative classification thresholds (Firth penalized logistic regression, n = 203).

| **Outcome and threshold** | **Events,**  **n (%)** | **Variables significantly associated** |
| --- | --- | --- |
| **Positive attitude** | | |
| Bloom 60%, ≥3.00 (primary) | 181 (89.2) | None |
| Bloom 80%, ≥4.00 | 116 (57.1) | Monthly income, all bands vs no personal income (OR 3.04 to 6.41) |
| Sample mean, ≥3.97 | 116 (57.1) | Monthly income, all bands vs no personal income (OR 3.04 to 6.41) |
| Sample median, ≥4.17 | 103 (50.7) | Monthly income, three of four bands (OR 3.66 to 5.27); postgraduate education (OR 2.15) |
| **Preconception preparation** | | |
| Any preparation, ≥1 (primary) | 89 (43.8) | None |
| Bloom 60%, ≥5.4 | 19 (9.4) | None |
| Bloom 80%, ≥7.2 | 5 (2.5) | Not interpretable, 5 events for 15 parameters |
| Sample mean, ≥1.59 | 72 (35.5) | None |

All models were adjusted for age group, nationality, education level, marital status, number of children, health insurance, employment status, and monthly income. Events per variable fell below 10 for all practice thresholds and for the stricter attitude thresholds, so these estimates should be interpreted with caution. The practice model at the Bloom 80% threshold produced extreme and unstable estimates owing to only five events and is not reported.

**Supplementary Appendix A.** English translation of the study questionnaire (the Arabic original administered to participants is provided below in appendix B).

**Questionnaire on the Knowledge of Women of Reproductive Age in Riyadh Regarding Pre-Conception Preparations**

This study aims to assess the knowledge and practice of women of reproductive age regarding pre-conception preparations in Saudi society. If you agree to participate in this study, please complete this questionnaire, which takes about 3 minutes. The data collected will be kept completely confidential, will not be used outside the scope of this research, and the questionnaire is completed anonymously. Thank you for your participation.

**Consent**

| **Question** | **Response options** |
| --- | --- |
| Do you agree to complete the questionnaire? | ☐ Yes ☐ No |

**A. Demographic Data**

| **Question** | **Response options** |
| --- | --- |
| 1. Age | ☐ 18–24 ☐ 25–29 ☐ 30–34 ☐ 35–45 |
| 2. Educational level | ☐ Secondary school or less / Diploma  ☐ University (bachelor’s degree)  ☐ Postgraduate education |
| 3. Marital status | ☐ Married ☐ Single ☐ Divorced ☐ Widowed |
| 4. What is your nationality? | ☐ Saudi ☐ Non-Saudi |
| 5. How many children do you have? | ☐ None ☐ 1–3 ☐ 4–5  ☐ 6–8 ☐ More than 8 |
| 6. Are you employed? | ☐ Yes ☐ No |
| 7. What is the monthly family income (SAR)? | ☐ No monthly income ☐ Less than 5,000  ☐ 5,000–9,999 ☐ 10,000–20,000  ☐ More than 20,000 |
| 8. Do you have health insurance? | ☐ Yes ☐ No |
| 9. What is your source of information regarding pre-conception preparations? | ☐ The internet  ☐ Family / friends  ☐ Study materials and courses  ☐ Health specialist (nurse, midwife, or physician who follows you during the reproductive period)  ☐ Multiple sources |

**B. Knowledge of Pre-Conception Preparations**

| **Question** | **Response options** |
| --- | --- |
| 1. Pre-conception preparations mean that a woman takes care of her health before pregnancy. | ☐ Yes ☐ No |
| 2. Pre-conception preparations include the assessment of risk factors for diseases before pregnancy. | ☐ Yes ☐ No |
| 3. Pre-conception preparations help to reduce fetal abnormalities in the uterus. | ☐ Yes ☐ No |
| 4. The pre-conception period is the period between planning for and starting pregnancy (about 3 months). | ☐ Yes ☐ No |
| 5. I adjust my lifestyle when planning for pregnancy in terms of regulating sleep, food and drink. | ☐ Yes ☐ No |
| 6. If I were a smoker, I would stop smoking when planning for pregnancy. | ☐ Yes ☐ No |
| 7. I undergo medical testing to make sure that I am not infected with HIV or viral hepatitis. | ☐ Yes ☐ No |
| 8. Taking folic acid prevents fetal malformations such as spina bifida. | ☐ Yes ☐ No |
| 9. Taking iron is needed if I have anaemia. | ☐ Yes ☐ No |
| 10. I stop strenuous physical exercise. | ☐ Yes ☐ No |
| 11. I stop consuming large amounts of caffeine. | ☐ Yes ☐ No |
| 12. I take care to avoid exposure to various occupational hazards when planning for pregnancy. | ☐ Yes ☐ No |
| 13. I make sure to reach my ideal weight when planning for pregnancy. | ☐ Yes ☐ No |
| 14. I eat a balanced and varied diet when planning for pregnancy. | ☐ Yes ☐ No |
| 15. I take my usual dose of vitamin D when planning for pregnancy. | ☐ Yes ☐ No |

**C. Practice and Application**

| **Question** | **Response options** |
| --- | --- |
| 1. Have you undertaken any pre-conception preparations? | ☐ Yes ☐ No |
| 2. Which type of pre-conception care did you undertake? (more than one option may be selected) | ☐ 1. Taking a folic acid supplement 3 months before pregnancy.  ☐ 2. Reaching an ideal weight (gaining or losing weight).  ☐ 3. Vaccination against (influenza or hepatitis B).  ☐ 4. Testing for sexually transmitted diseases.  ☐ 5. Quitting smoking / shisha / vaping.  ☐ 6. Improving the quality of food and increasing its variety.  ☐ 7. Exercising regularly (two to five times per week).  ☐ 8. Screening for chronic diseases such as diabetes, hypertension, etc.  ☐ 9. Stopping the consumption of ready-made (fast) foods. |

**D. Attitudes and Beliefs Toward Pre-Conception Preparations**

*(Five-point Likert scale; one answer per statement)*

| **Question** | **Response options** |
| --- | --- |
| 1. Pre-conception preparations have no positive effect on the outcome of pregnancy. | ☐ 1 Strongly agree ☐ 2 Agree ☐ 3 Neutral  ☐ 4 Disagree ☐ 5 Strongly disagree |
| 2. Pre-conception preparations are not important for women of reproductive age. | ☐ 1 Strongly agree ☐ 2 Agree ☐ 3 Neutral  ☐ 4 Disagree ☐ 5 Strongly disagree |
| 3. Clinics dedicated to pre-conception preparations are a luxury service that is not needed. | ☐ 1 Strongly agree ☐ 2 Agree ☐ 3 Neutral  ☐ 4 Disagree ☐ 5 Strongly disagree |
| 4. There is not enough time to undertake pre-conception preparations. | ☐ 1 Strongly agree ☐ 2 Agree ☐ 3 Neutral  ☐ 4 Disagree ☐ 5 Strongly disagree |
| 5. Do you think that only mothers at high risk of pregnancy complications start pre-conception care when planning for pregnancy? | ☐ 1 Strongly agree ☐ 2 Agree ☐ 3 Neutral  ☐ 4 Disagree ☐ 5 Strongly disagree |
| 6. Only mothers with a history of fetal malformation should follow pre-conception care. | ☐ 1 Strongly agree ☐ 2 Agree ☐ 3 Neutral  ☐ 4 Disagree ☐ 5 Strongly disagree |

**E. Barriers and Obstacles Preventing Saudi Women from Accessing Pre-Conception Care**

1. What are the barriers or obstacles that prevent or hinder women from accessing pre-conception care in Saudi Arabia? (open-ended response)

**Supplementary Appendix B*:*** Original Arabic questionnaire (as administered to participants)

**استبانة قياس مدى معرفة السيدات في عمر الإنجاب في الرياض بتحضيرات ما قبل الحمل**

تهدف هذه الدراسة الى تحديد مدى معرفة و ممارسة السيدات في عمر الإنجاب بتحضيرات ما قبل الحمل، في المجتمع السعودي. اذا كنت توافقين على المشاركة في الدراسة، يرجى إكمال هذا الاستبيان لمدة 3 دقائق. علما بأن البيانات التي سيتم أخذها ستكون سرية تماما، ولن يتم استخدامها خارج نطاق البحث، كذلك تتم تعبئة البحث بدون اسم. أشكركم على مشاركتكم.

**ا- هل توافق على تعبئة الاستبيان؟**

| **السؤال** | **الإجابة** |
| --- | --- |
| هل توافق على تعبئة الاستبيان؟ | ☐ نعم ☐ لا |

**أ- البيانات الديموغرافية:**

| **السؤال** | **الإجابة** |
| --- | --- |
| 1. العمر | ☐ 18-24 ☐ 25-29 ☐ 30-34 ☐ 35-45 |
| 2. المستوى التعليمي | ☐ ثانوي او اقل/ دبلوم ☐ جامعي ☐ دراسات عليا |
| 3. الحالة الاجتماعية | ☐ متزوجة ☐ اعزب ☐ مطلقة ☐ ارملة |
| 4. ماهي جنسيتك؟ | ☐ سعودية ☐ غير سعودية |
| 5. كم عدد ابناءك؟ | ☐ لا يوجد ☐ 1-3 ☐ 4-5  ☐ 6-8 ☐ أكثر من 8 |
| 6. هل انت موظفة؟ | ☐ نعم ☐ لا |
| 7. ماهو الدخل الشهري للعائلة؟ | ☐ لا يوجد دخل شهري ☐ أقل من 5000  ☐ 5000-9999 ☐ 10000-20000  ☐ أكثر من 20000 |
| 8. هل لديك تأمين صحي؟ | ☐ نعم ☐ لا |
| 9. مصدر المعلومات لديك لاستعدادات ما قبل الحمل | ☐ الانترنت  ☐ العائلة/ الاصدقاء  ☐ مواد دراسية و دورات  ☐ اخصائي صحة: ممرضة او قابلة او طبيبة تتابعك خلال مرحلة عمر الإنجاب  ☐ مصادر متعددة |

**ب- المعرفة باستعدادات ما قبل الحمل (Knowledge)**

| **السؤال** | **الإجابة** |
| --- | --- |
| 1- استعدادات ما قبل الحمل تعني عناية السيدة بجسمها صحيا قبل الحمل | ☐ نعم ☐ لا |
| 2- استعدادات ما قبل الحمل تتضمن تقييم عوامل الخطر للاصابة بالامراض قبل الحمل | ☐ نعم ☐ لا |
| 3- استعدادات ما قبل الحمل تساعد في تقليل تشوهات الجنين داخل الرحم | ☐ نعم ☐ لا |
| 4- معنى فترة ما قبل الحمل هي الفترة بين التخطيط وبدء الحمل (حول 3 اشهر) | ☐ نعم ☐ لا |
| 5- أضبط حياتي عند التخطيط للحمل من ناحية تنظيم النوم و الاكل و الشرب | ☐ نعم ☐ لا |
| 6- اذا كنت مدخنة، اتوقف عن التدخين عند التخطيط للحمل | ☐ نعم ☐ لا |
| 7- أفحص طبيا للتأكد من عدم الاصابة بالايدز او الكبد الوبائي | ☐ نعم ☐ لا |
| 8- تناول الفوليك اسيد لمنع الاصابة بتشوهات الاجنة مثل الصلب المفتوح | ☐ نعم ☐ لا |
| 9- تناول الحديد اذا كنت مصابة بالانيميا | ☐ نعم ☐ لا |
| 10- اتوقف عن التمارين الرياضية الشديدة | ☐ نعم ☐ لا |
| 11- اتوقف عن تناول الكافيين بكميات كبيرة | ☐ نعم ☐ لا |
| 12- انتبه لعدم التعرض للمخاطر المهنية المختلفة عند التخطيط للحمل | ☐ نعم ☐ لا |
| 13- أحرص على الوصول للوزن المثالي عند التخطيط للحمل | ☐ نعم ☐ لا |
| 14- أتناول غذاء متوازنا متنوعا عند التخطيط للحمل | ☐ نعم ☐ لا |
| 15- أتناول جرعاتي الاعتيادية من فيتامين د عند التخطيط للحمل | ☐ نعم ☐ لا |

**ج. الممارسة و التطبيق (Practice)**

| **السؤال** | **الإجابة** |
| --- | --- |
| 1- هل قمت باستعدادات ما قبل الحمل؟ | ☐ نعم ☐ لا |
| 2- نوع رعاية ما قبل الحمل الذي تم اتخاذه: (يمكن اختيار اكثر من خيار) | ☐ 1. تناول مكملات حمض الفوليك قبل 3 أشهر من الحمل.  ☐ 2. الوصول للوزن المثالي (زيادة وزن او تنزيل وزن).  ☐ 3. التحصين ضد (الأنفلونزا، والتهاب الكبد B).  ☐ 4. فحص الأمراض المنقولة عن طريق الاتصال الجنسي.  ☐ 5. الاقلاع عن التدخين/ الارجيلة او الفيب.  ☐ 6. تحسين نوعية الغذاء و زيادة التنوع فيه.  ☐ 7. ممارسة التمارين الرياضية (مرتين الى خمس مرات اسبوعيا).  ☐ 8. فحص الأمراض المزمنة مثل السكري و الضغط وغيره.  ☐ 9. التوقف عن تناول الاغذية الجاهزة. |

**د. التوجهات و الأفكار تجاه استعدادات ما قبل الحمل (Attitudes)**

| **السؤال** | **الإجابة** |
| --- | --- |
| 1. استعدادات ما قبل الحمل ليس لها أي تأثير ايجابي على نتيجة الحمل | ☐ 1 اتفق بشدة ☐ 2 اتفق ☐ 3 محايد  ☐ 4 لا اتفق ☐ 5 لا اتفق بشدة |
| 2. استعدادات ما قبل الحمل ليست مهمة للسيدات في عمر الانجاب | ☐ 1 اتفق بشدة ☐ 2 اتفق ☐ 3 محايد  ☐ 4 لا اتفق ☐ 5 لا اتفق بشدة |
| 3. العيادات المتخصصة باستعدادات ما قبل الحمل تعتبر رفاهية لا حاجة لها | ☐ 1 اتفق بشدة ☐ 2 اتفق ☐ 3 محايد  ☐ 4 لا اتفق ☐ 5 لا اتفق بشدة |
| 4. ليس هناك وقتا كافيا لاتخاذ استعدادات ما قبل الحمل | ☐ 1 اتفق بشدة ☐ 2 اتفق ☐ 3 محايد  ☐ 4 لا اتفق ☐ 5 لا اتفق بشدة |
| 5. هل تعتقدين أن الأمهات المعرضات لمخاطر الحمل هم اللواتي يبدأن فقط رعاية ما قبل الحمل عند التخطيط للحمل | ☐ 1 اتفق بشدة ☐ 2 اتفق ☐ 3 محايد  ☐ 4 لا اتفق ☐ 5 لا اتفق بشدة |
| 6. الامهات اللواتي عانين من تشوه الاجنة هم اللواتي يجب ان يتبعن رعاية ما قبل الحمل فقط؟ | ☐ 1 اتفق بشدة ☐ 2 اتفق ☐ 3 محايد  ☐ 4 لا اتفق ☐ 5 لا اتفق بشدة |

**هـ. الحواجز والعقبات التي تمنع النساء السعوديات من الوصول إلى الرعاية السابقة للحمل**

1- ما هي الحواجز او العقبات التي تمنع او تعيق النساء من الوصول لرعاية ما قبل الحمل في السعودية؟ (إجابة مفتوحة)
